# Pontine Myelinolysis Caused by Hypovolemic Hypernatremia

**DOI:** 10.1155/2020/4079098

**Published:** 2020-09-07

**Authors:** D. O. Beraldo, S. B. C. P. Duarte, R. B. Santos, C. G. Mendes, M. P. Silveira, A. S. Neto, M. M. Silva, L. G. Oliveira, A. V. Bonfim, A. A. Teixeira, R. A. Teixeira

**Affiliations:** ^1^Hospital Renascentista, Pouso Alegre, MG, Brazil; ^2^Escola Paulista de Medicina—UNIFESP, São Paulo, Brazil; ^3^Instituto do Coração—FMUSP, São Paulo, Brazil

## Abstract

*Introduction*. Central pontine myelinolysis is characterized by the occurrence of acute demyelinating lesions of cells in the pons secondary to abrupt oscillations of serum osmolarity. Its exact incidence is not well defined, but studies show a prevalence of 0.25 to 0.5% in the general population, 2.5% in the intensive care unit, and up to 10% in patients with risk factors, such as chronic liver disease and hepatic transplantation, alcoholism, malnutrition, diuretic therapy, electrolyte imbalance, hypoglycemia, and hyperglycemia. *Case Report*. A 70-year-old white female with extranodal diffuse large B-cell non-Hodgkin's lymphoma (extensive mass on the left anterior chest wall), stage IVA, developed pontine myelinolysis secondary to hypovolemic acute hypernatremia, which occurred due to diarrhea caused by chemotherapy (rituximab, cyclophosphamide, doxorubicin, and vincristine). *Discussion.* Pontine myelinolysis occurs most often due to the rapid correction of chronic hyponatremia. But here, we describe a case of the disease secondary to the occurrence of hypovolemic acute hypernatremia in a patient with a hematological malignancy under treatment, who was on chronic treatment with thiazide diuretics and who presented with other electrolyte disturbances as risk factors for the development of pontine myelinolysis.

## 1. Introduction

Central pontine myelinolysis (CPM) was first described in 1959 by Adams et al. as a disease that affected alcoholic and malnourished patients and was characterized by acute demyelinating lesion of pontine cells secondary to abrupt oscillations of serum osmolarity [1]. The term myelinolysis was intentionally used to highlight the predominance of noninflammatory lesions to the myelin as opposed to other neuronal structures [1]. It is within the spectrum of the osmotic demyelination syndrome (ODS), which is divided into CPM and extrapontine myelinolysis, which in turn are distinguished by involvement of the central region of the pons or regions outside it, respectively [2, 3].

The exact incidence of the disease is not well defined, but studies show a prevalence of 0.25 to 0.5% in the general population, 2.5% in the intensive care unit, and up to 10% in patients undergoing liver transplantation [4–7]. Some groups are more predisposed to the development of ODS, such as alcoholics; malnourished individuals; those with liver disease; chronic users of diuretics; patients with other electrolyte disturbances (hypokalemia, hypophosphatemia, and hypernatremia); individuals with hypoglycemia and hyperglycemia, central and nephrogenic diabetes insipidus, and adrenal insufficiency; those with Wilson's disease, celiac disease, and psychiatric conditions such as psychogenic polydipsia, and women during pregnancy and puerperium [8–15].

The main etiopathogenic mechanism of ODS is the rapid correction of hyponatremia, especially in the scenario of hyponatremia with chronic evolution. A low serum sodium concentration leads to serum hyposmolarity and the consequent passage of free water into the intracellular environment of the cells of the central nervous system, which promotes cellular edema. In cases of chronic evolution, cells adapt and lose osmoles (electrolytes and organic osmoles, such as myoinositol, taurine, glutamine, glutamate, creatinine, and glycerophosphorylcholine) and free water as a compensatory mechanism. In the scenario of rapid correction of hyponatremia, there is an acute increase in serum osmolarity and consequent dehydration of cells of the central nervous system, leading to noninflammatory demyelinating lesions and apoptosis [9, 16–20].

This case report describes the occurrence of CPM related to hypovolemic acute hypernatremia secondary to diarrhea. It is a rare cause of ODS, which is often related to the correction of hyponatremia.

## 2. Case Report

A 70-year-old white female was diagnosed in June 2017 with extranodal diffuse large B-cell non-Hodgkin's lymphoma (extensive mass on the left anterior chest wall), stage IVA. Chemotherapy was initiated with the R–CHOP regimen (rituximab, cyclophosphamide, doxorubicin, and vincristine) in the same month, performing the first two cycles using a Port-a-Cath catheter, without complications and with a substantial reduction of the left anterior thoracic mass. Comorbidities included hypertension for 3 years, hemorrhoidal disease (conservative treatment), bilateral knee arthrosis with total left knee arthroplasty 3 years earlier, and esophageal hiatus hernia (conservative treatment). Associated with this was a history of an imagining finding of an asymptomatic pulmonary thromboembolism during the staging of the hematological malignancy and history of poliomyelitis at 8 years of age with motor sequelae in the lower limbs (with unaided walking ability maintained). The patient had no history of smoking or drinking, and medications consisted in chronic use of chlortalidone 25 mg once daily, omeprazole 40 mg once daily, rivaroxaban 20 mg once daily, bromopride 10 mg once daily, and sulfamethoxazole/trimethoprim 800/160 mg 3x a week.

The patient underwent the third cycle of chemotherapy in September 2017, and 10 days later, she began to suffer from general malaise, hyporexia, prostration, lesions of the oral mucosa, acute voluminous diarrhea without pathological products, and fever (38.3°C). She was admitted to the intensive care unit of Hospital Renascentista with a diagnosis of high-risk febrile neutropenia with no defined infectious focus (MASCC score of 11 points), severe mucositis of the gastrointestinal tract with considerable oral lesions and diarrhea, prerenal acute renal failure, and severe pancytopenia after chemotherapy. Physical examination showed that the patient was dehydrated (3/4+), prostrate, less communicative and febrile (38.3°C), had poor peripheral perfusion with blood pressure 80/50 mmHg and heart rate 110 bpm, and had substantial oral mucositis, with no other changes in the general and neurological examination. Laboratory tests showed the following results: hemoglobin 6.7 g/dL, leukocytes 270 cells/mm^3^ (20% neutrophils), platelets 8000 cells/mm^3^, creatinine 2.6 mg/dL, urea 194 mg/dL, fasting glucose 82 mg/dL, sodium 164 mEq/L, magnesium 1.4 mEq/L, phosphorus 2.2 mg/dL, potassium 2.2 mEq/L, AST 23 IU/L, ALT 20 IU/L, albumin 2.1 g/dL, bilirubins normal, coagulation tests normal, arterial lactate 31 mg/L, pH 7.21, HCO3 16 mEq/L, chest X-ray normal, urinalysis normal, urine culture negative, and blood cultures negative.

**Figure 1:**
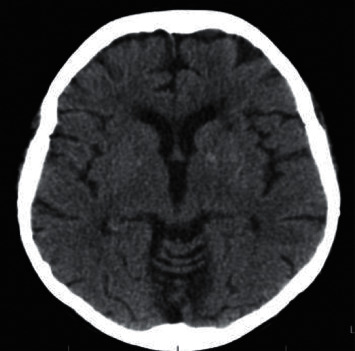
Cranial computed tomography showing previous left lacunar stroke.

**Figure 2:**
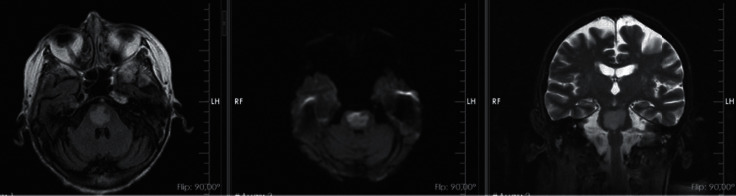
Cranial magnetic resonance showing findings in the pons suggestive of pontine myelinolysis (sequences: axial T2, diffusion, and coronal T2).

Immediate empirical antibiotic treatment was initiated with piperacillin-tazobactam and vancomycin due to high-risk febrile neutropenia with multiple risk factors for Gram-positive infection (mucositis, long-term invasive device, acute neurological disorder, multiple organ dysfunction, and hypotension), in addition to hemodynamic support of sepsis, volume resuscitation, transfusion support, and correction of water-electrolyte imbalance. She evolved with hemodynamic improvement, resolution of fever, improvement of renal function, and improvement of hypomagnesemia, hypokalemia, and hypophosphatemia, with progressive and slow sodium correction (<0.5 mEq/L/hour) until normalization after several days. However, considerable diarrhea and pancytopenia followed, which began to improve after two weeks of hospitalization. From a neurological point of view, despite clinical and laboratory improvement, she maintained prostration and developed a fluctuating lowered level of consciousness, grade I paresis of upper limbs and grade III paresis of lower limbs, mild dysarthria, upper and lower limb hyporeflexia, no signs of pyramidal release, and normal brain stem reflexes and ocular motricity. A complementary investigation was performed with cranial computed tomography, which showed only a previous small left lacunar stroke (Figure 1); electroencephalogram, which showed changes suggestive of toxic-metabolic damage to the central nervous system; cerebrospinal fluid analysis, which was normal; and cranial magnetic resonance, which revealed changes suggestive of pontine myelinolysis (Figure 2).

**Figure 3:**
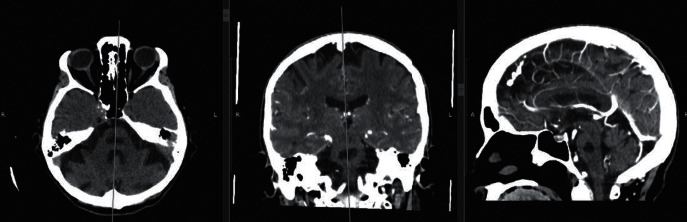
Cranial computed tomography showing a hypoattenuating focal area in the central region of the pons (sequences: axial, coronal, and sagittal).

The patient continued with clinical and laboratory improvement, with complete resolution of pancytopenia, water-electrolyte imbalance, renal dysfunction, and diarrhea. She had a slow neurological improvement and was discharged with normal level of consciousness and progressive recovery of motor deficits. After two years, her neurological status was the same as before the central pontine myelinolysis event, and her cranial computed tomography revealed a hypoattenuating focal area in the central region of the pons (Figure 3).

## 3. Discussion

The clinical picture of CPM includes a variety of neurological manifestations, which include flaccid quadriparesis with subsequent evolution to the spastic phase, dysarthria and dysphagia (pseudobulbar palsy), oculomotor and pupillary changes, and lowered level of consciousness. In some situations, locked-in syndrome may occur. These neurological changes are due to the involvement of the pontine structures to different degrees, particularly the base of the pons (corticobulbar and corticospinal tracts) and tegmentum of the pons [9]. The symptoms usually appear 2 to 7 days after the electrolyte changes [21], and there is no apparent relationship between the severity of the clinical condition and the initial sodium values, the rate of sodium correction, and the presence of associated comorbidities [8].

The diagnosis of CPM should be considered in cases of acute onset of neurological symptoms related to sodium imbalance, especially rapid correction of hyponatremia (>0.5 mEq/L/hour), where it is confirmed by imaging of the central nervous system, with prominence for magnetic resonance, whose findings are bilateral hypersignal in the central region of the pons in the T2 and FLAIR sequences and hyposignal in the T1 sequence [22]. Radiological changes usually appear days after clinical manifestations, and the diffusion sequence in MRI has been shown to be effective for an earlier diagnosis [23, 24].

The approach in CPM consists mainly in preventive measures based on the correct handling of the correction of sodium imbalance since there are no well-established treatments. Some approaches have been studied in recent years, with emphasis on immunoglobulin therapy or plasmapheresis, which possibly promote remyelinization and reduction of myelotoxicity. Rodriguez et al. described 3 cases of pontine myelinolysis treated with immunoglobulin and observed clinical improvement after treatment [25]. Also, Saner et al. evaluated immunoglobulin and plasmapheresis therapy in a renal transplant patient and also found an improvement in neurological status [26].

Pontine myelinolysis occurs most often due to rapid correction of chronic hyponatremia [27]. However, there are rare reports in the literature of CPM secondary to other osmolal disorders (hyperglycemia and hypoglycemia) [28, 29], in addition to cases with normal serum sodium [30] or in the presence of rapid onset hypernatremia in patients with risk factors for OSD (34). Kilinç et al. described an episode of pontine myelinolysis with normal serum sodium in a 34-year-old male patient on chronic renal dialysis [30]. Additionally, Varanda et al. reported a case of pontine myelinolysis caused by multifactorial hypernatremia in a female patient with liver cirrhosis who was a regular alcohol drinker, which is in agreement with our findings [31].

## 4. Conclusion

We describe here a case of CPM related to the occurrence of hypovolemic acute hypernatremia secondary to diarrhea in a patient with a hematological malignancy under treatment, presenting with the risk factors such as chronic use of a thiazide diuretic and the presence of other electrolyte disturbances (hypophosphatemia, hypokalemia, and hypomagnesemia).
